# The impact of antiretroviral therapy on iron homeostasis and inflammation markers in HIV-infected patients with mild anemia

**DOI:** 10.1186/s12967-017-1358-6

**Published:** 2017-12-19

**Authors:** Eugenia Quiros-Roldan, Francesco Castelli, Paola Lanza, Chiara Pezzoli, Marika Vezzoli, Inflammation in HIV Study Group, Alessandro Montanelli, Giuseppina Ruggeri, Eugenio Monti, Andrea Bonito, Alice Ferraresi, Emanuele Focà, Ilaria Izzo, Giorgio Biasiotto, Isabella Zanella

**Affiliations:** 1 University Department of Infectious and Tropical Diseases, University of Brescia and Spedali Civili General Hospital, Brescia, Italy; 2 University Department of Infectious and Tropical Diseases, Spedali Civili General Hospital, Brescia, Italy; 3 Department of Molecular and Translational Medicine, University of Brescia, Brescia, Italy; 4 Laboratory of Clinical Chemistry, Department of Diagnostics, Civic Hospital of Brescia, Brescia, Italy

**Keywords:** cART, HIV, Inflammation, Iron, Hepcidin, Anemia

## Abstract

**Background:**

Anemia is frequent during HIV infection and is predictive of mortality. Although cART has demonstrated to reduce its prevalence, several patients still experience unresolved anemia. We aimed to characterize iron homeostasis and inflammation in HIV-infected individuals with mild anemia in relation to cART.

**Methods:**

In this retrospective cohort study, HIV-infected patients with mild anemia, CD4^+^ cells > 200/mm^3^ at baseline, maintaining virological response for 12 months after cART starting were selected within the Standardized Management of Antiretroviral Therapy Cohort (MASTER) cohort. Several inflammation and immune activation markers and iron homeostasis indexes were measured in stored samples, obtained at cART initiation (T0) and 12 months later (T1). Patients were grouped on the basis of hemoglobin values at T1: group A (> 13 g/dl) and B (< 13 g/dl). Wilcoxon rank sum test was used to compare biomarker values. Pearson correlation coefficients were calculated for all variables.

**Results:**

cART improved CD4^+^ and CD8^+^ cell counts and their ratio, but this effect was significant only in group A. Only these patients had mild iron deficiency at T0 and showed higher transferrin and lower percentage of transferrin saturation than patients of group B, but differences disappeared with cART. cART decreased inflammation in all patients, but group B had higher levels of all markers than group A, reaching statistical significance only for IL-8 values at T1 (16 vs 2.9 pg/ml; p = 0.017). Hepcidin and IL-6 levels did not show significant differences between groups. Hemoglobin levels both at T0 and T1 did not correlate with any marker.

**Conclusions:**

Baseline mild anemia in HIV-infected patients cannot always be resolved with durable efficient cART, possibly due to residual inflammation or immune activation rather than unbalanced iron homeostasis. Further research is needed on cytokine profiling to understand the mechanisms that induce anemia in HIV with suppressive cART.

**Electronic supplementary material:**

The online version of this article (10.1186/s12967-017-1358-6) contains supplementary material, which is available to authorized users.

## Background

Chronic inflammation contributes to the development of anemia in both general population [1, 2] and human immunodeficiency virus (HIV)-infected people [3, 4]. Anemia is the most common haematological abnormality in HIV seropositive patients and is predictive of HIV-associated morbidity and mortality, independently of CD4^+^ count [4–10].

Anemia in HIV infection is a complex mechanism. HIV itself produces chronic immune activation and inflammation and, under this inflammatory conditions, dietary iron is blocked from enterocyte release, whereas circulating iron is redistributed into cellular storage locations including macrophages. Additional HIV-associated consequences, including suppression of several hematopoietic cells [11] and opportunistic infections through inflammation further contribute to anemia during HIV infection. Although combination anti-retroviral therapy (cART) has demonstrated to reduce the prevalence of anemia in many HIV-infected patients, a considerable proportion of those patients experiences unresolved anemia or develop anemia after its initiation [12, 13]. Many studies have focused on the impact of cART on inflammation and immune activation markers [3, 12], but there are few prospective studies dealing with the association between anemia, iron metabolism and inflammation markers in HIV-infected individuals before and after cART [14].

The pathophysiology of anemia during infections is multifactorial, resulting from the effects of inflammatory cytokines, such as IL-6 and possibly other cytokines involved in host defense and leading to hepcidin-induced hypoferremia. The pro-inflammatory cytokine IL-6 is a dominant regulator of hepatic hepcidin production in bacterial infections and other inflammatory conditions, but IL-6 concentrations are often only mildly elevated in viral infections. Data on hepcidin levels in HIV infected patients are rarely reported [15–21].

Alterations of iron homeostasis are common in chronic inflammatory and infectious diseases [22]. In fact, prevalence of anemia among HIV-infected patients is high [3, 6] and it is frequently resolved after cART initiation [23, 24]. Sometimes, however, anemia can persist in HIV-chronically-infected patients, even after HIV replication was suppressed with cART, and it has been associated with elevated levels of IL-6, CRP and D-dimer [3] and with monocyte activation [25]. This condition is considered as anemia of chronic inflammation.

This study was designed to characterize iron homeostasis and inflammation in relation to anemia at HIV diagnosis, and after the decline of virus titers in plasma due to cART introduction in a group of HIV-infected patients with mild anemia and CD4^+^ cells > 200/mm^3^.

## Methods

### Patients

We conducted a retrospective cohort study. Patients were recruited from the HIV-infected patients of the Standardized Management of Antiretroviral Therapy Cohort (MASTER cohort) followed in the University Department of Infectious and Tropical Diseases of the University of Brescia [26]. Anemia was defined based on WHO hemoglobin cutoffs [27]. We retrospectively selected all Caucasian HIV-infected male patients with the following characteristics: (i) hemoglobin (Hb) value between 9.5 and 13 g/dl and CD4^+^ cell count > 200/mm^3^ before cART initiation (T0); (ii) plasma HIV RNA < 37 copies/ml after 6 months of first cART; (iii) maintained cART response (plasma HIV RNA < 37 copies/ml at 6 and 12 months of stable cART) (T1 = 12 months); (iv) available plasma samples either at T0 and T1, stored at − 80 °C. Patients were classified into 2 groups: group A, with Hb < 13 g/dl at T0 and Hb > 13 g/dl at T1 and group B, with Hb < 13 g/dl both at T0 and T1. Exclusion criteria were: (i) severe anemia (Hb < 8 g/dl), (ii) acute HIV-infection, (iii) severe concomitant diseases (opportunistic infections, cancer) at T0 or during the study period, congenital disorders of Hb synthesis, (iv) also female gender was a criterion for exclusion to reduce the confounding of iron loss.

### Assessment of inflammatory and iron metabolism markers

Plasma samples at T0 and T1 were tested for levels of predefined inflammatory and iron homeostasis markers. Serum C reactive protein (CRP), iron and transferrin (TF) were quantified using the Dimension Vista 5 Siemens automated analyzer, using commercially available reagents (Siemens Healthcare GmbH, Erlangen, Germany). The percentage of transferrin saturation (%TS) was calculated on the basis of iron and TF values. Serum ferritin levels were measured using the Abbott Architect automated analyzer with the Abbott Architect Ferritin Assay (Abbott Laboratories, Chicago, IL, US). Hb levels were determined using the Beckman Coulter Unicell DxH800analyzer (Beckman Coulter, Brea, CA, US). IL-2, IL-3, IL-4, IL-5, IL-7, IL-8, IL-10, IL-18, interferon-γ (IFN-γ), tumor necrosis factor-α (TNF-α), tumor necrosis factor-γ (TNF-γ), Macrophage inflammatory protein-1-α (MIP-1-α), macrophage inflammatory protein-1-β (MIP-1-β), monocyte chemotactic protein 1 (MCP-1) and granulocyte–macrophage colony-stimulating factor (GM-CSF) serum levels were measured using the Myriad RBM Cytokine MAP A panel (Myriad RBM, Austin TX, US). Commercial ELISA kits were used to quantify serum levels of IL-6, IL-22 (Cloud-Clone Corporation, Houston, TX, USA) and hepcidin (DRG Diagnostic, Marburg, Germany), according to the manufacturer’s protocols.

### Statistical analysis

Student’s *t* test (for age) and *χ* ^*2*^ test (for HCV coinfection, tobacco and intravenous drug use) were used to compare patients of group A and B for demographic and epidemiological characteristics. Quantitative variables were compared at baseline (T0) and 12 months later (T1) using Wilcoxon rank sum tests for paired samples in all samples (n = 18), group A (n = 10) and group B (n = 8). The same variables were analyzed comparing group A vs group B, both at T0 and T1, using Wilcoxon rank sum tests for independent samples. *p* < 0.05 was considered to be statistically significant. Pearson correlation coefficients were computed to determine possible associations between biomarkers, resulting in a symmetric correlation matrix (Additional file 1) (highlighted in bold the coefficients major of 0.7 in absolute value, when p values are < 0.05). In heatmap graphical representation, values contained in a datamatrix, previously standardized with mean 0 and standard deviation 1, were represented with colors. In detail, low (negative), mean (near to 0), and high (positive) values were represented with blue, yellow and red, respectively. Heatmaps place similar values near each other according to the clustering algorithm used in the analysis which is based on the Euclidean distance. Hence, we obtained two dendrograms which were added to the heatmap on *x*- and *y*-axis, respectively.

### Ethics statement

The study was conducted in accordance with the Declaration of Helsinki and the principles of Good Clinical Practice. All patients provided written informed consent to include their clinical and biological data in the MASTER database [22]. All patients signed the informed consent for archiving a plasma sample at baseline and every 6 months for scientific purposes. The study was approved by the Ethical Committee of the Civic Hospital of Brescia (Coordinating Centre) and of all the participant Centers [26].

## Results

The studied population consisted of 18 HIV-1-infected treatment-naïve patients with mild anemia (Hb value between 9.5 and 13 g/dl) at time of initiating cART (T0). After cART, all patients had plasma HIV-1 RNA levels consistently < 37 copies/ml, as measured by commercial assays at 6, 9 and 12 months of treatment. Ten patients increased Hb values above 13 g/dl at T1 and then they were retrospectively included in group A (median Hb values: T0 = 12.70 g/dl; T1 = 14.25 g/dl; p = 0.0020), while 8 patients remained anemic at T1 and were retrospectively included in group B (median Hb values: T0 = 11.85 g/dl; T1 = 11.95 g/dl; p = 0.3828). The characteristics of the population are shown in Table 1. Among patients of group A, none used intravenous drugs or had hepatitis C virus (HCV) chronic infection and only 30% were habitual smokers. Among patients of group B, HCV chronic infection was present in 62.5% (5 out 8) of patients, but any HCV coinfected patient was under HCV treatment, 75% (6 out 8) had acquired HIV-infection by intravenous drug use and 100% were smokers.

**Table 1:** Demografic and epidemiological characteristics of patients

	Total (n = 18)	Group A (n = 10)	Group B (n = 8)	*p *value
Age (years) (mean ± SD)	42.8 ± 8.6	45.6 ± 9.6	39.3 ± 6.0	0.1221
HCV infected (n%)	5 (27.8%)	0	5 (62.5%)	0.0033
Smokers (n%)	11 (61.1%)	3 (30%)	8 (100%)	0.0025
Intravenous drug users	6 (33.3%)	0	6 (75%)	0.0019

Student’s *t* test was used to compare age and *χ* ^*2*^ test to compare HCV coinfection, tobacco and intravenous drug use

As expected, cART improved CD4^+^ cell count, and CD4^+^/CD8^+^ ratio (T0 vs T1 median values). This effect was statistically significant for patients from group A, but not for those from group B (Table 2).

**Table 2:** Comparison of T0 and T1 biomarker values

Variables	Median (min–max) All patients (n = 18)		*p * value*	Median (min–max) Group A (n = 10)		*p * value*	Median (min–max) Group B (n = 8)		*p * value*	Median (min–max) T0		*p * value**	Median (min–max) T1		*p * value**
	T0	T1		T0	T1		T0	T1		Group A	Group B		Group A	Group B	
CD4^+^	410.00 (10.00–831.00)	566.00 (252.00–1026.00)	0.0305	410.00 (10.00–831.00)	672.50 (334.00–1026.00)	0.0078	429.50 (106.00–697.00)	550.50 (252.00–733.00)	0.9453	410.00 (10.00–831.00)	429.50 (106.00–697.00)	0.9626	672.50 (334.00–1026.00)	550.50 (252.00–733.00)	0.3154
CD8^+^	1036.00 (423.00–2063.00)	962.50 (234.00–3100.00)	0.0833	986.50 (550.00–1865.00)	970.50 (409.00–3100.00)	0.0781	1090.00 (423.00–2063.00)	854.00 (254.00–1859.00)	0.4609	986.50 (550.00–1865.00)	1090.00 (423.00–2063.00)	0.7984	970.50 (409.00–3100.00)	854.00 (254.00–1859.00)	0.5726
CD4^+^/CD8^+^	0.60 (0.00–1.10)	0.64 (0.16–1.97)	*0.0032*	0.60 (0.00–1.00)	0.70 (0.16–1.97)	*0.0039*	0.55 (0.20–1.10)	0.61 (0.22–1.38)	0.2500	0.60 (0.00–1.00)	0.55 (0.20–1.10)	0.8087	0.70 (0.16–1.97)	0.61 (0.22–1.38)	0.9654
Viremia	23,270.00 (25.00–863,800.00)	< 37.00	*0.0005*	34,657.00 (4146.00–135,000.00)	< 37.00	*0.0039*	12,350.00 (25.00–863,800.00)	< 37.00	*0.0225*	34,657.00 (4146.00–135,000.00)	12,350.00 (25.00–863,800.00)	0.1672	< 37.00	< 37.00	1.0000
Hemoglobin (g/dl)	12.35 (9.50–12.90)	13.25 (10.80–15.70)	*0.0008*	12.70 (10.90–12.90)	14.25 (13.20–15.70)	*0.0020*	11.85 (9.50–12.80)	11.95 (10.80–12.90)	0.3828	12.70 (10.90–12.90)	11.85 (9.50–12.80)	0.0895	14.25 (13.20–15.70)	11.95 (10.80–12.90)	*0.0004*
IL-6 (pg/ml)	5.74 (3.29–9.83)	4.58 (2.91–11.91)	0.8317	6.11 (3.73–9.83)	4.97 (3.49–11.91)	0.8457	4.52 (3.29–8.50)	4.23 (2.91–7.05)	1.0000	6.11 (3.73–9.83)	4.52 (3.29–8.50)	0.2743	4.97 (3.49–11.91)	4.23 (2.91–7.05)	0.2133
IL-8 (pg/ml)	11.00 (2.90–418.00)	2.90 (2.90–119.00)	*0.0418*	9.65 (2.90–60.00)	2.90 (2.90–23.00)	0.0756	19.50 (2.90–418.00)	16.00 (2.90–119.00)	0.3828	9.65 (2.90–60.00)	19.50 (2.90–418.00)	0.5333	2.90 (2.90–23.00)	16.00 (2.90–119.00)	*0.0169*
IL-18 (pg/ml)	335.50 (145.00–868.00)	228.50 (111.00–808.00)	*0.0092*	282.50 (145.00–466.00)	149.50 (119.00–808.00)	0.1235	428.00 (145.00–868.00)	348.00 (111.00–533.00)	*0.0391*	282.50 (145.00–466.00)	428.00 (145.00–868.00)	0.0618	149.50 (119.00–808.00)	348.00 (111.00–533.00)	0.0816
MIP-1-β (pg/ml)	175.50 (83.00–687.00)	183.00 (30.00–981.00)	0.6164	175.50 (113.00–286.00)	125.50 (30.00–981.00)	0.4922	176.00 (83.00–687.00)	192.00 (72.00–620.00)	0.9453	175.50 (113.00–286.00)	176.00 (83.00–687.00)	0.8937	125.50 (30.00–981.00)	192.00 (72.00–620.00)	0.4232
MCP-1 (pg/ml)	166.00 (43.50–606.00)	124.00 (43.50–288.00)	*0.0097*	160.00 (43.50–606.00)	116.50 (43.50–288.00)	0.0972	166.00 (93.00–489.00)	131.50 (43.50–167.00)	*0.0360*	160.00 (43.50–606.00)	166.00 (93.00–489.00)	0.8239	116.50 (43.50–288.00)	131.50 (43.50–167.00)	0.8570
IL-22 (pg/ml)	16.50 (8.00–70.00)	16.00 (4.00–28.00)	0.4936	14.00 (8.00–70.00)	13.00 (4.00–22.00)	0.4406	19.00 (9.00–33.00)	19.50 (11.00–28.00)	1.0000	14.00 (8.00–70.00)	19.00 (9.00–33.00)	0.1177	13.00 (4.00–22.00)	19.50 (11.00–28.00)	0.1085
Hepcidin (ng/ml)	2.06 (0.50–22.93)	2.67 (0.33–11.38)	0.8650	4.41 (0.50–22.93)	2.71 (0.33–11.38)	0.7695	1.84 (0.52–3.87)	2.46 (0.72–6.92)	0.5469	4.41 (0.50–22.93)	1.84 (0.52–3.87)	0.2301	2.71 (0.33–11.38)	2.46 (0.72–6.92)	0.3740
Ferritin (ng/ml)	171.00 (9.00–415.00)	90.00 (6.00–280.00)	*0.0120*	192.50 (9.00–415.00)	80.00 (6.00–220.00)	*0.0488*	149.00 (16.00–303.00)	9.50 (58.00–280.00)	0.1953	192.50 (9.00–415.00)	149.00 (16.00–303.00)	0.5148	80.00 (6.00–220.00)	9.50 (58.00–280.00)	0.5938
Hepcidin/ferritin	0.02 (0.01–0.06)	0.03 (0.01–0.15)	0.0539	0.03 (0.02–0.06)	0.05 (0.01–0.15)	0.0645	0.02 (0.01–0.04)	0.01 (0.01–0.07)	0.3828	0.03 (0.02–0.06)	0.02 (0.01–0.04)	0.2031	0.05 (0.01–0.15)	0.01 (0.01–0.07)	0.0545
Iron (µg/dl)	73.00 (32.00–112.00)	71.00 (24.00–160.00)	0.6790	54.00 (32.00–91.00)	71.00 (24.00–160.00)	0.2408	85.50 (42.00–112.00)	70.50 (42.00–156.00)	0.3828	54.00 (32.00–91.00)	85.50 (42.00–112.00)	*0.0455*	71.00 (24.00–160.00)	70.50 (42.00–156.00)	0.7618
Transferrin (mg/dl)	195.00 (122.00–269.00)	201.50 (133.00–311.00)	0.7403	203.00 (144.00–269.00)	115.50 (148.00–311.00)	0.5533	184.50 (122.00–230.00)	184.50 (133.00–225.00)	0.7260	203.00 (144.00–269.00)	184.50 (122.00–230.00)	0.3154	115.50 (148.00–311.00)	184.50 (133.00–225.00)	0.0619
Transferrin saturation (%)	23.89 (9.31–55.99)	29.83 (7.35–55.21)	0.5509	21.41 (9.31–28.52)	29.83 (7.35–45.25)	0.2754	34.28 (16.34–55.99)	30.02 (16.69–55.21)	0.6406	21.41 (9.31–28.52)	34.28 (16.34–55.99)	*0.0085*	29.83 (7.35–45.25)	30.02 (16.69–55.21)	0.6334

Biomarker values were compared at T0 and T1 for all patients, patients of group A, patients of group B and patients of group A vs group B. Median values and minimum/maximum values are reported for each group. Wilcoxon rank sum test for paired samples (*p *value*) and Wilcoxon rank sum test for independent samples (*p *value**) were used for comparison. In italics *p* > 0.05

### Iron metabolism

Patients of group A, who resolved anemia with cART, had mild iron deficiency at T0, but a trend to the increase of serum iron levels with anti-retroviral therapy was observed (from 54 mg/dl at T0 up to 71 mg/dl at T1). Conversely, in patients of group B, plasma iron levels were within the reference values (> 60 mg/dl for adult male), despite of anemia and significantly higher than in patients of group A at T0 (85.5 mg/dl vs 54 mg/dl; p = 0.0455) and iron values did not significantly changed with cART (T0 = 85.5 mg dl and at T1 = 70.5 mg/dl, p = 0.3828). Further, while TF levels did not significantly change in both groups with cART, patients of group A had higher TF values than patients of group B at both times, although the difference was not statistically significant (203 mg/dl vs 184.5 mg/dl at T0; p = 0.3154; and 215.5 mg/dl vs 184.5 mg/dl at T1; p = 0.0619). The %TS was significantly lower in patients of group A than in patients of group B at T0 (21.41% vs 34.28%; p = 0.0085), but the difference disappeared with cART (29.83% vs 30.02%; p = 0.6334) (Table 2).

### Inflammation markers

In general, cART decreased values of several inflammatory markers in all patients. But, while in patients of group A, the decrease was significant only for ferritin (192.5 ng/ml vs 80 ng/ml; p = 0.0488), in patients of group B it was significant for IL-18 (428 pg/ml vs 348 pg/ml; p = 0.0391) and MCP-1 (166 pg/ml vs 131 pg/ml; p = 0.0360). Further, we found that all inflammatory markers were always higher in patients of group B than in patients of group A, although reaching statistical significance only for IL-8 values at T1 (16 vs 2.9 pg/ml; p = 0.017) (Table 2).

Only in patients of group B, we observed that cART increased MIP-1-β and hepcidin values, although the increase did not reach the statistical significance (176 pg/ml vs 192 pg/ml; p = 0.9453; and 1.84 vs 2.46 ng/ml; p = 0.5469, respectively). Hepcidin and IL-6 levels did not show significant differences between groups, but a trend toward higher levels in group A vs group B were observed (hepcidin at T0: 4.41 ng/ml vs 1.84 ng/ml; p = 0.2301; hepcidin at T1: 2.71 ng/ml vs 2.46 ng/ml; p = 0.3740; IL-6 at T0: 6.11 pg/ml vs 4.52 pg/ml; p = 0.2743; IL-6 at T1: 4.97 pg/ml vs 4.23 pg/ml; p = 0.2133). Interestingly, the ratio hepcidin/ferritin increased with cART in patients of group A (0.03 vs 0.05; p = 0.0645), but decreased in patients of group B (0.02 vs 0.01; p = 0.3828) (Table 2).

**Fig. 1:**
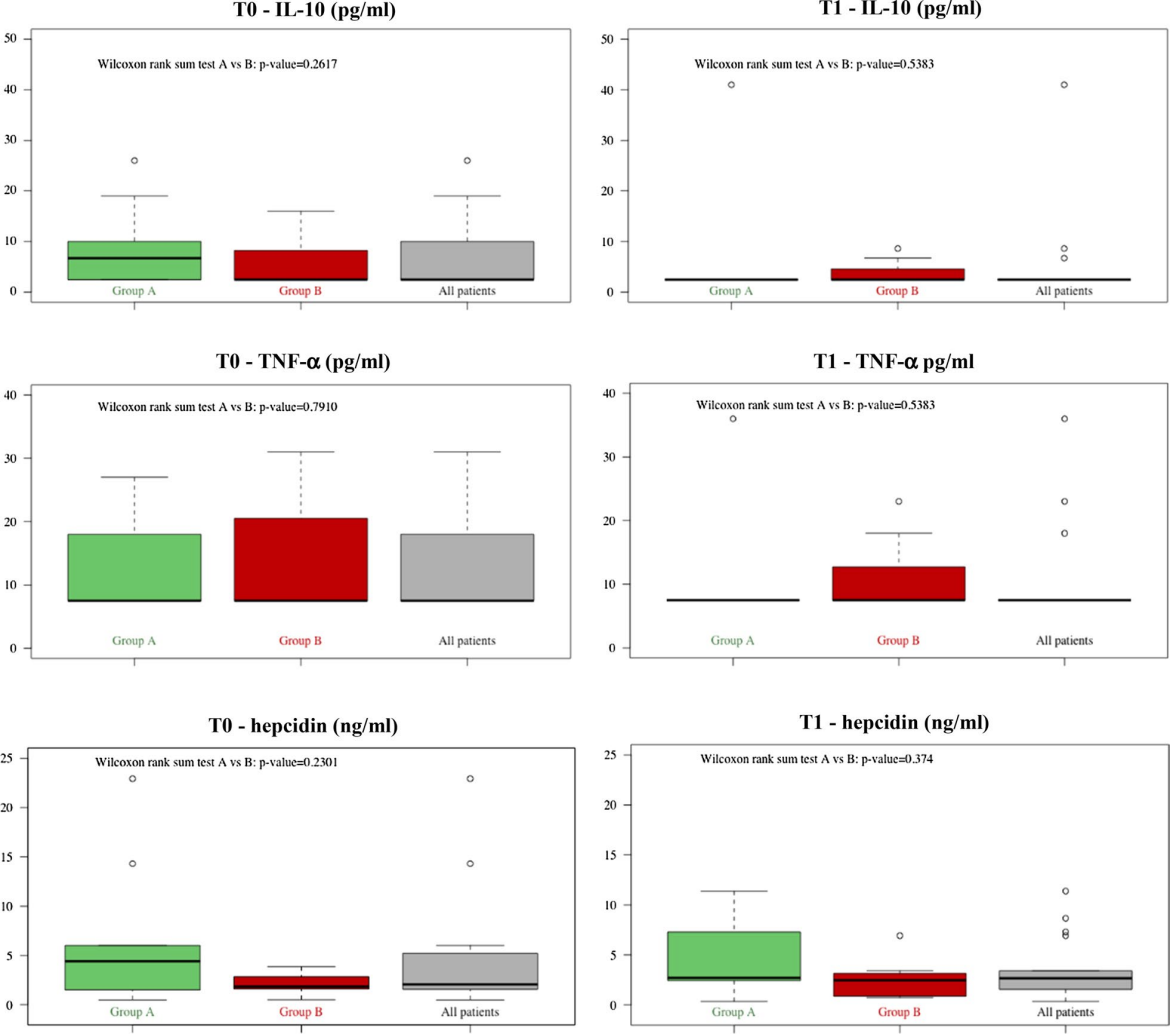
Biomarker levels in HIV-infected patients. Serum levels of IL-10, tumor necrosis factor α (TNF-α) and hepcidin in HIV-infected patients at baseline (T0) and after 12 months (T1) of combination anti-retroviral therapy (cART) in group A, group B and all patients. Wilcoxon rank sum test for independent samples was used to compare median values of patients of group A vs patients of group B. Outliers were identified by means of circles. *p* values are also reported

Serum levels of some markers were below the detection limit of the assays (CRP, IL-2, IL-3, IL-4, IL-5, IL-7, GM-CSF, TNF-β and IFN-γ) in most patients and were not included in the study, while for most samples IL-10, TNF-α and MIP-1-α were detectable and interestingly decreased below the detection limit at T1 only in patients from group A (Fig. 1).

### Correlation matrix and heatmaps representation

**Fig. 2:**
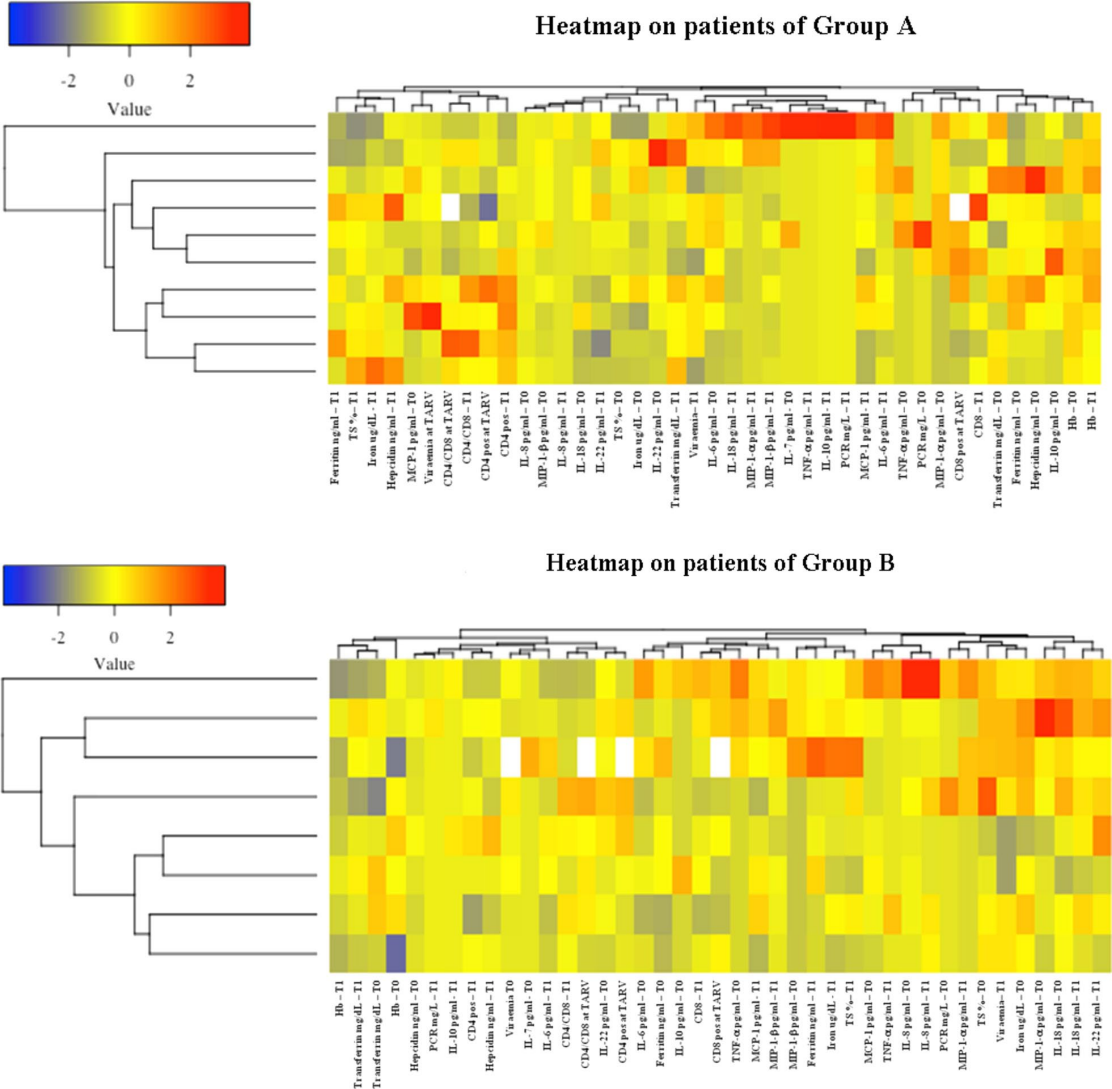
Heatmap analysis. Heatmap analysis on biomarker levels relative to patients in group A and in group B

We did not found linear relationship between Hb or iron levels when all 18 patients were considered together with hepcidin, any marker of inflammation, CD4^+^, CD8^+^ cell count or CD4^+^/CD8^+^ ratio (Additional file 1). We also performed the heatmap analysis to identify possible clusters or pathways among variables (Fig. 2). Interestingly, only in group B, iron was clustered among inflammatory markers both at T0 and T1, but not with Hb, TF, hepcidin and IL-6.

## Discussion

Here, we describe that baseline mild anemia in HIV-infected patients cannot always be resolved with durable efficient cART. In general, cART decreased values of inflammatory markers and ameliorated iron metabolism in all patients. But HIV-infected patients with anemia that is resolved with cART had lower levels of IL-8, IL-18, IL-22, MIP-1-β and MCP-1 and higher IL-6 and hepcidin levels than patients with persistent anemia, either at T0 and T1. On the other hand, HIV-infected patients with anemia that is not resolved with cART are frequently coinfected with HCV (62%), and at baseline they have higher values of iron, %TS and IL-18 and a trend toward lower levels of hepcidin and IL-6, compared to those patients in whom anemia was resolved by cART. Furthermore, patients with persistent anemia had minor immune recovery.

Several studies in HIV-infected patients have found an inverse association between hepcidin and absolute CD4^+^ cell count [17, 21], suggesting that elevated hepcidin may be ascribed to advanced disease stage and elevated levels of inflammation. Most recently, Rosado-Sanchez et al. [28] observed that high levels of IL-6, CRP, high CD4^+^ cell turnover and regulatory T cell (Treg) frequency are already present prior to initiation of cART in HIV-infected patients with poorer CD4^+^ cell recovery, suggesting a role of inflammation on CD4^+^ cell homeostasis. In HIV-infected patients the chronic exposure to other antigens or pathogens, like cytomegalovirus (CMV) or HCV, may also contribute to persistent inflammation or immune activation [29–31] and clinical events [32, 33]. In cART-treated HIV-infection with minimal CD4^+^ cell defects, HCV seems not to be associated with cellular immune activation, but it may rather contribute to immune exhaustion [30]. In our study that includes HIV-infected patients from an Italian cohort with mild anemia and median CD4^+^ cells > 400/mm^3^ at baseline, cART significantly increased CD4^+^ cell count in patients of group A (none HIV/HCV infected), but not in group B (62% of them was HIV/HCV coinfected).

cART initiation consistently leads to decrease in most systemic inflammatory markers, T cell indices and monocyte activation, albeit rarely to levels comparable to those of HIV-uninfected individuals [34]. This chronic state of inflammation/immune activation, that it is established during HIV-infection as a result of the complex interactions between viral and host factors, may be associated to clinical consequences despite of efficient cART. Increased values of IL-18 have been associated with clinical cART failure in HIV-infected patients [35] and high level of IL-8 has been associated with lower number of CD4^+^ cell count and HIV disease progression in children [36]. Patients of group B in our study, who did not recover from anemia, had indeed higher levels of IL-8 and IL-18.

Many potential mechanisms for this chronic inflammatory state have been proposed: HIV itself, translocated microbial products, other chronic concomitant pathogens, including CMV and HCV, and lipids [37, 38]. Despite of cART success, HIV/HCV coinfected patients have poorer prognosis and more rapid progression of liver disease, probably due to the excessive inflammation/immune activation in HIV/HCV coinfection, compared to either HIV or HCV mono-infection [39, 40].

Interactions between HIV and HCV in respect to inflammation and iron metabolism are complex and poorly known. Distinct patterns of iron homeostasis occur during different viral infections. Acute HIV infection causes a decrease of serum iron that is accompanied by increase in hepcidin, CRP, IL-10, IL-18 and TNF-α. Levels remained elevated in cART treated patients, in spite of undetectable HIV replication and Hb normalization. Interestingly, hepcidin up-regulation or hypoferremia were not evidenced during acute HCV or HBV infection [19]. In contrast, a recent study in chronically HCV-infected patients has showed that liver disease activity has a negative impact on erythropoiesis with compensatory higher but blunted EPO responses [41].

Data are emerging about differences between HIV monoinfected and HIV/HCV coinfected patients in levels of markers of inflammation and iron metabolism dysregulation. In vitro co-stimulation of HepG2 cells with HCV-E2 and HIV-gp120 proteins induces a potent proinflammatory response with the production of IL-8 [42] and it is suggested that in vivo IL-8 could be responsible for the local inflammatory changes that induce fibrosis in HCV-infected patients and the severe liver damage observed in HCV/HIV co-infected patients [43]. Higher plasma levels of IL-6, IL-8, IL-18 and MIP-1-β have been recently observed in HIV/HCV coinfected patients than in both monoinfections. It has been hypothesized that high levels of some of these cytokines could be responsible for the increased incidence and progression of inflammatory illness in HIV/HCV chronically coinfected patients, even in patients on cART with undetectable HIV RNA [30, 44]. Here, we observed that cART decreased IL-8, IL-18 and MCP-1 in all patients, however their levels remained higher in group B than in group A. cART has been described to induce slow fibrosis progression in HIV/HCV-coinfected patients with successful HIV suppression, but progression remains accelerated compared with HCV-monoinfected patients [45]. Indeed, recent studies have shown that hepcidin levels is reduced in both liver and serum of patients with HCV infection, which may contribute to pathological liver iron storage and elevated serum ferritin and iron levels [46–48].

We found that IL-8 level was higher in group B (that included most of patients with chronic HCV infection) than in group A and, although cART decreased its level in both groups, it remained significantly higher in group B. These patients were anemic with normal values of serum iron and %TS, significantly higher than those of patients of group A at T0, and, despite the control of HIV replication with cART for more than 6 months.

Our study has some limitations. First, the study was performed in a limited number of patients. Second, we do not know if there were differences in time of infection between the two groups, despite the fact that both groups showed similar time from diagnosis and the mean of CD4^+^ cells between groups was similar. Third, the present study was not designed to analyze differences in inflammatory cytokine or iron marker levels between HIV/HCV and HIV monoinfected patients, but, casually, most patients of group B were HIV/HCV infected [39, 49]. The longitudinal design of this study, in contrast with other studies comparing several groups in a cross-sectional way, confers it an important strength. The participants in this cohort had sustained virologic suppression, below the limits of detection of commercial assays, mitigating the potential effects of virologic failure or transient viremia on measures of anemia or inflammation, that might be observed in less-adherent individuals. Another strength of the present study is the high number of inflammatory markers tested.

## Conclusions

Additional inflammation other than HIV-mediated can be responsible for the persistent mild chronic anemia and for the slower CD4^+^ and CD4^+^/CD8^+^ increase in patients with sustained plasmatic HIV suppression by cART. Understanding the mechanisms that mediate the inflammation/immune activation in HIV with suppressive cART is a challenging issue. Profiling the cytokines will plausibly enable identification of factors that could complement the information from viral load and CD4^+^ cell counts in the management of the disease and therapeutic interventions.

## Additional file

**Additional file 1.** Pearson correlation analysis. Pearson correlation analysis between variables considered in this study (Hb, HIV-related factors, markers of iron homeostasis, of immune activation and inflammation). In bold Pearson correlation coefficients with *rho* > |0.7| and *p values* < 0.05 are shown.

## Abbreviations

cART: combination anti-retroviral therapy
CRP: C reactive protein
CMV: cytomegalovirus
GM-CSF: granulocyte–macrophage colony-stimulating factor
Hb: hemoglobin
HCV: hepatitis C virus
HIV: human immunodeficiency virus
IL-2: interleukine-2
IL-3: interleukine-3
IL-4: interleukine-4
IL-5: interleukine-5
IL-6: interleukine-6
IL-7: interleukine-7
IL-8: interleukine-8
IL-10: interleukine-10
IL-18: interleukine 18
IL-22: interleukine-22
IFN-γ: interferon-γ
MCP-1: monocyte chemotactic protein 1
MIP-1-α: macrophage inflammatory protein-1-α
MIP-1-β: macrophage inflammatory protein-1-β
%TS: percentage of transferrin saturation
TF: transferrin
Treg: regulatory T cell
TNF-α: tumor necrosis factor-α
TNF-β: tumor necrosis factor-β
